# Essential medicines wastage assessment in the Solomon Islands

**DOI:** 10.1371/journal.pgph.0000181

**Published:** 2022-02-01

**Authors:** Ibrahim Dadari, Effua Usuf

**Affiliations:** 1 United Nations Children’s Fund (UNICEF) Pacific, UN Joint Presence, ANZ Haus, Honiara, Solomon Islands; 2 Medical Research Council Unit The Gambia @London School of Hygiene and Tropical Medicine, Fajara, The Gambia; Bahauddin Zakariya University, PAKISTAN

## Abstract

The World Health Organization (WHO) regularly updates its list of Essential medicines. However, the use of these medicines in the Solomon Islands is less well described. We assessed supplies, prescriptions, and stocks of zinc, oral rehydration salt (ORS), vitamin A, and albendazole in six provinces of the Solomon Islands for 2017 and 2018. We also conducted a stocktake of available medicine supplies at the point of data collection. We found that quantities of drugs supplied were in excess of the prescriptions and stock records at the facilities were inadequate. There were expired drugs at the facilities. Out of the 20 health facilities with available data, 11 (55%), 3 (15%), and 1 (5%) had expired stock of ORS, albendazole and vitamin A respectively. No expired zinc tablets were recorded. A revision of the medicines stock management system is necessary to adequately quantify essential medicine wastage and improve the stock management in the Solomon Islands.

## Introduction

Since 1977, WHO has provided a list of essential medicines–these are “medicines that satisfy the priority health care needs of a population” [1]. In 2007, the list was adapted to cover medicines for children specifically. According to WHO, about 2 billion of the world’s population cannot access essential medicines [2]. Most of these are people living in developing countries [3]. Though the reasons for limited access are varied, increasing drug cost and irrational use remain a challenge in many settings [4]. An evaluation of essential medicines availability and drug wastage rates in a small zone in Ethiopia observed significant wastage rates with revenue loss estimated at 1,343,974 Ethiopian Birr (USD 29, 500) [5]. Another cross-sectional study in the Oromia region found wastage rates of 6.3% for oral rehydration salts and up to 39.1% for anti-infective agents [6]. With a more efficient supply chain and medicines usage, resource loss to waste could impact positively on public health service delivery. Essential medicines wastage also affects more advanced countries. Annual drug wastage was estimated to cost £300 million in England in the last decade [7], and in the United States, consumers waste $418 billion due to suboptimal use of medicines [8]. A cross-section of pharmacists in the Gulf perceived that waste minimizing activities were important, however not all were implemented in their daily practice [9].

In general, wastage from prescribed medications is any medication that remains unused or expires anywhere along the medicines supply chain [10]. Data on usage and wastage of essential medicines are lacking, which are needed to guide setting acceptable limits for essential medicines wastage. Understanding the problem is vital to develop strategies to minimize the wastage of these ‘essential’ drugs. Therefore, studies to evaluate how these medicines are used are needed.

The Solomon Islands is a country in the South Pacific with an estimated population of 653,248 as of 2017, 50.5% of which are children and youth less than 19 years of age [11]. The country lies northwest of Vanuatu and east of Papua New Guinea (PNG), having more than 900 Islands. Honiara city council is the capital, and there are nine other provinces in the country. The Solomon Islands Ministry of Health and Medical Services (MHMS) pharmacy division, based at the national medical stores (NMS), manages all medicines and medical supplies in the country, which makes the same available to all public health facilities free of charge. Essential medicines and supplies like vaccines are delivered through a three-tier system from the NMS to the 18 second-level medical stores (SLMS) and finally, to over 350 service delivery points or health facilities [12]. The country uses an electronic medicines inventory management platform (mSupply) at the NMS and in some SLMS. Paper-based records are used across all clinics, which seemingly is stronger for vaccines than other medicines and supplies. The MHMS and partners were interested in identifying bottlenecks in essential medicines as part of comprehensive primary health care (PHC) strengthening. Alongside an assessment on vaccine wastage, this project reviewed the stock of four essential medicines—Zinc, oral rehydration salt (ORS), Vitamin A, and Albendazole at health facilities in the Solomon Islands. The four medicines were selected considering disease burden–high diarrhea and malnutrition rates, and the potential ease of measurement. The Solomon Islands has unique challenges relating to its size and geography; therefore, it is essential to estimate the current wastage rates and where they occur to guide the establishment of national targets and set up systems to mitigate and monitor wastage regularly.

## Methods

### Setting

Essential medicines, vaccines, and supplies are warehoused at the NMS from where supplies are sent to the Secondary Level Medical Stores (SLMS), which in turn supplies most primary level health facilities, including hospitals, Area Health Centers (AHCs), Rural Health Clinics (RHCs) and Nurse Aid Posts (NAP). Few health facilities such as the national referral hospital and some clinics in Honiara city council and Guadalcanal province get supplies directly from the NMS.

The study was conducted across six provinces as described previously in another submitted paper, which described the assessment of vaccine wastage in the Solomon Islands with the estimated wastage rates for the various vaccine antigens used in the country. This paper distills the unique challenges of essential medicines in the assessment. Briefly, we purposively selected the provinces to include the two largest (Malaita and Guadalcanal) and two smallest (RenBel and Temotu) provinces and the Honiara city council, which hosts the NMS, and the Western province due to its strategic location at the Solomons-PNG border. We randomly selected SLMSs, AHCs and RHCs, and NAPs within each province, with the total number proportionate to the number of facilities in that province.

### Data collection

We applied a structured questionnaire at 22 health facilities at the same time when a vaccine wastage survey was being conducted. To assure validity and reliability, developed questionnaires were reviewed with subject matter specialists from both the MHMS and UNICEF, followed by a field-testing of the questionnaire in select urban and rural clinics from which final adjustments were made. We trained a team of data collectors who are public health and nursing graduates from the national university (in-class and practical) and formed into groups of two each per province, with senior health workers assigned as supervisors over the teams. Data were available and collected on the number of prescriptions of interest from the 2017 and 2018 outpatient registers. We also reviewed the medical supplies and drug order forms for the quantities of essential medicines received from the NMS or SLMS and supplied to other service delivery level facilities. We checked the essential medicines stock at each facility and physically counted the stock at the site on data collection between July and August 2019.

We supplemented these data at the facilities with the data obtained from the MHMS District Health Information Software 2 (DHIS2) fed from monthly reports on the doses of albendazole and vitamin A prescribed. zinc and ORS are not captured in the monthly health activity reports submitted by the facilities to the DHIS2. We also reviewed the mSupply at the NMS for the quantity of medicines distributed to the facilities.

### Analysis

For each of the medicines of interest, we described the prescription patterns and compared these to the quantities received from the medical stores. The reasons for discarding or not using the essential medicines were explained where possible.

### Ethics statement

The Solomon Islands Health Research and Ethics Review Board approved this study with approval certificate number HRE030/19. For the essential medicines’ wastage assessment bit, no individual consent was required as the survey involved retrieving stock information from the registers and conducting a physical stock take. However, we sought permission from the health workers to access the records. No patient identifiers in our data, all data retrieved and used in this study were fully anonymized before being accessed.

## Results

### Supplies

There was wide variability in the quantities of the essential medicines supplied to the health facilities. Not more than 20,000 zinc tablets (20mg) were supplied to each SLMSs in 2017 and 2018 except for one facility that received about 50,000 tablets in 2018. At the other primary level facilities, less than 2500 tablets were supplied, except one facility that received >10,000 in 2017 and about 5000 in 2018.

Supplies for the four drugs to the facilities is shown in S1 Fig.

### Prescriptions

The prescription patterns also varied widely among facilities and between 2017 and 2018. The maximum prescribed medicines were both in the capital, Honiara, in 2017 and 2018. Prescriptions for vitamin A were small compared to the others, perhaps because vitamin A, unlike the others, is mainly prescribed to under-fives. The quantity supplied was more than the quantity prescribed at all facilities Table 1 and S2 Fig. The discrepancies are likely to be due in part to incomplete data.

**Table 1:** Quantity supplied from the National Medical Stores and number of prescriptions at the facilities for the four essential medicines, 2017 and 2018.

Medicine(tablets/sachets)	Year	Quantity Supplied	Number of prescriptions*
**Albendazole**	2017	366,000	2,767
	2018	107,000	5,525
**ORS**	2017	60,500	3,359
	2018	31,300	2,041
**Vitamin A**	2017	165,000	376
	2018	123,000	933
**Zinc**	2017	93,900	2,239
	2018	130,500	1,453

Units of the drugs used at the facilities were—Zn 20mg, Albendazole 200/400mg, Vitamin A 100,000 IU.

*Prescriptions per person.

### Essential medicines stock management at the facilities

Actual stock records of the essential medicines of interest were only available at two facilities, and these data were incomplete. At least one facility had stock out for one of the four medicines (Table 2).

**Table 2:** Essential medicines stock (physical count) at the facilities on the visit day.

Medicine	Number of facilities n (%)		Total Quantity of expired drugs (all facilities)
	Out of stock	Expired stock	
	n/N (%)	n/N (%)	
Zinc	5/20(25)	0	0
ORS	12/20(60)	11/20 (55)	2795
Vitamin A	1/20(5)	1/20 (5)	1100
Albendazole	2/20(10)	3/20 (15)	3350

Units of the drugs at the facilities were- Zn 20mg, Albendazole 200/400mg, Vitamin A 100,000 IU.

Data not available at two facilities.

We found a total of 2795 ORS sachets that had expired in April 2019 from seven facilities and an additional 1100 sachets of ORS that will expire in October 2019. 1100 vitamin A capsules at one facility had expired, and an additional 57,240 capsules that would expire in September 2019 were present in 14/20 facilities. Three facilities had 2000, 850, and 500 expired tablets of albendazole, respectively. There were no expired zinc tablets.

## Discussions

Our attempt to describe essential medicine wastage gives credence to an integrated approach to improving immunization and the essential medicines supply chain. However, we found substantial data gaps with no apparent trends in the supplies or prescription patterns of the reviewed essential drugs. Recording and stock management were poor at the facilities. In comparison, vaccine data were generally more available at the health facilities. This provides a premise for integrated essential medicines and vaccines supply chain strengthening to piggy-back essential medicines stock management improvement on the advances made already in the immunization supply chain while also making the immunization supply chain even stronger for vaccines and supplies security at the facility levels.

More so, looking at some of the essential medicines stock availability at the health facility levels, for instance, the quantity of zinc tablets available seems highly suboptimal with the Solomon Islands having thousands of diarrhea cases annually and probably most needing zinc supplementation for 10 to 14 days as recommended [13]. This could have resulted from low demand or pull-factor with the NMS not procuring and distributing enough zinc, also with zinc stockouts less than half of those reported for ORS. However, zinc supplies increased by more than 50% in 2018 relative to 2017 values. ORS seems to have a more considerable demand with more stockouts reported. Although these should be interpreted with caution considering the data gaps noticed across facilities.

A qualitative study in Ethiopia listed poor communication between stores and dispensing units, non-compliance to first expired and first out principle, weak monitoring systems as factors contributing to medicines waste [14]; not surprisingly, the pharmacists interviewed in that study reported that stock out was a significant challenge. In another study in South Africa, more than 80% of expired medicines were listed as essential medicines [15]. Expiration was the only type of wastage observed in our study. Additionally, more than half of the facilities saw large quantities of Vitamin A close to the expiry date. The absence of stock records at the facilities is likely a contributing factor; therefore, proper stock management will minimize this type of wastage.

Though there were considerable discrepancies in the number of drugs supplied compared to the number of prescriptions, the wastage is likely high in this setting. A significant limitation of the project was the incompleteness of the data, which indicates the magnitude of the challenge and the need for an assessment to understand supplies and usage of drugs better. Medicines stock management at the facilities needs to be reviewed as only two facilities had some record. The rollout of the msupply to the facilities should cover medicines. Future studies may extend to other sectors of the supply chain, including consumers.

## Supporting information

S1 Data Medicines wastage data. (XLSX)

S1 Fig Quantity of essential medicines supplied for Zinc, ORS, Albendazole and Vitamin A. (DOCX)

S2 Fig Prescription patterns for Zinc, ORS, Albendazole and Vitamin A. (DOCX)
