# Supplementary material for: Essential medicines wastage assessment in the Solomon Islands
Source: PLOS Glob Public Health. 2022 Feb 1;2(2):e0000181. doi: 10.1371/journal.pgph.0000181 (PMC10022118; doi:10.1371/journal.pgph.0000181)
Supplement: S1 Fig — (DOCX) [file pgph.0000181.s002.docx]

Supplementary figure 1

*Quantity of essential medicines supplied (A1, A2) Zinc, (B1, B2) ORS (C1, C2) Albendazole and (D1, D2) Vitamin A*

A1

A2

B1

B2

C1

C2

D1

D2
