# Supplementary material for: Essential medicines wastage assessment in the Solomon Islands
Source: PLOS Glob Public Health. 2022 Feb 1;2(2):e0000181. doi: 10.1371/journal.pgph.0000181 (PMC10022118; doi:10.1371/journal.pgph.0000181)
Supplement: S2 Fig — (DOCX) [file pgph.0000181.s003.docx]

Supplementary figure 2

*Prescription patterns* *(A1, A2) Zinc, (B1, B2) ORS (C1, C2) Albendazole and (D1, D2) Vitamin A*

A 1

A2

B 1

B2

C 1

C2

D 1

D2
